# Supplementary material for: The zinc-finger transcription factor Blimp1/Prdm1 is required for uterine remodelling and repair in the mouse
Source: Nat Commun. 2025 Jan 31;16:1220. doi: 10.1038/s41467-025-56511-8 (PMC11785775; doi:10.1038/s41467-025-56511-8)
Supplement: Supplementary file 1 — Supplementary Information [file 41467_2025_56511_MOESM1_ESM.pdf]

Supplementary Information

The zinc-finger transcription factor Blimp1/Prdm1 is required for uterine remodelling and repair in the mouse

Maria-Eleni Xypolita, Mubeen Goolam, Elizabeth K Bikoff, Elizabeth J Robertson, and Arne W Mould

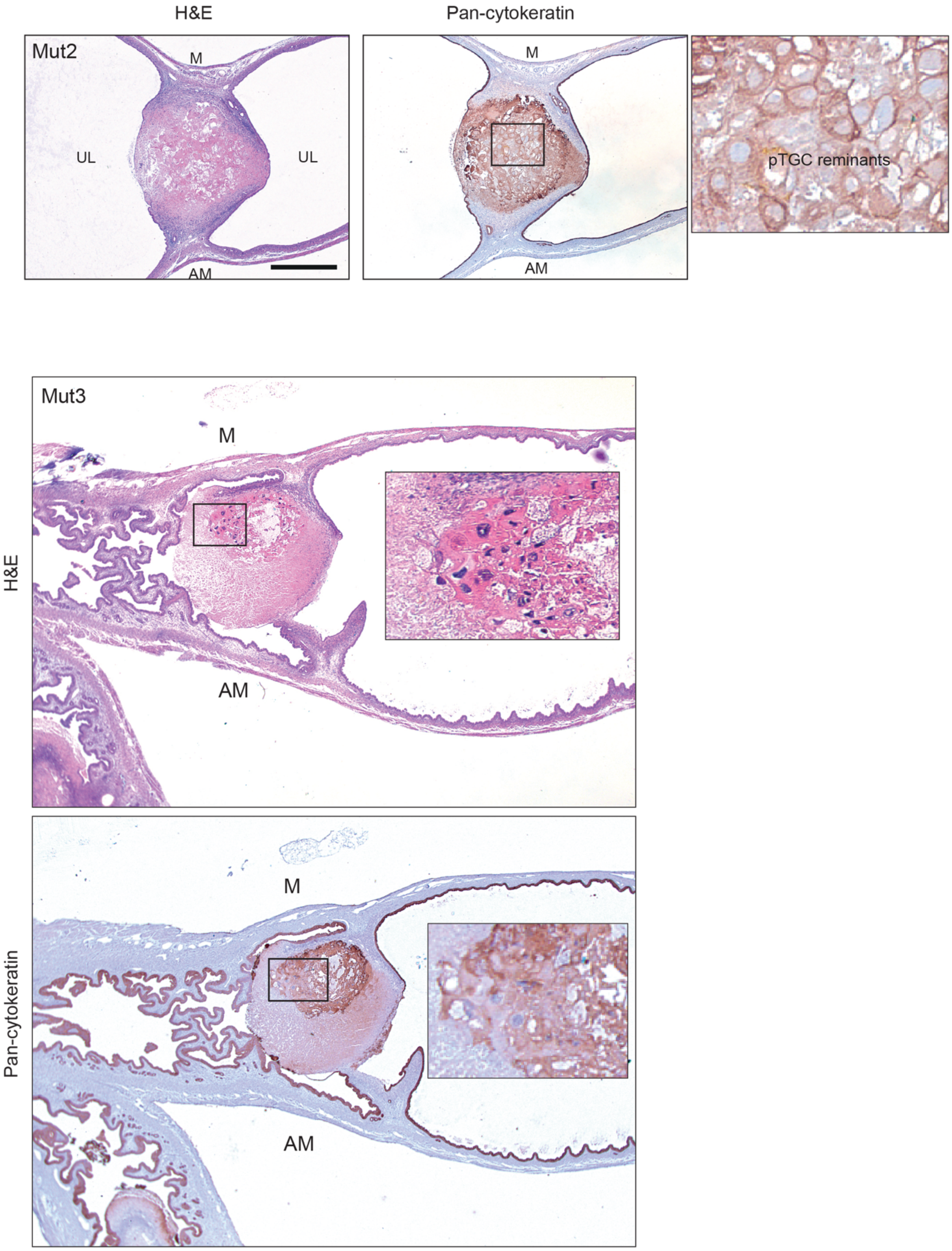

Supplementary Fig. 1 Blimp1 mutants display impaired uterine remodelling at post-partum day 5. Additional examples of H&E and pan-cytokeratin stained uterine occlusions present in Blimp1 mutant uteri (n=3) at PP5 showing persistence of TGCs. Mut= mutant, UL = uterine lumen, M = mesometrial, AM = antimesometrial. Scale bar =1 mm.

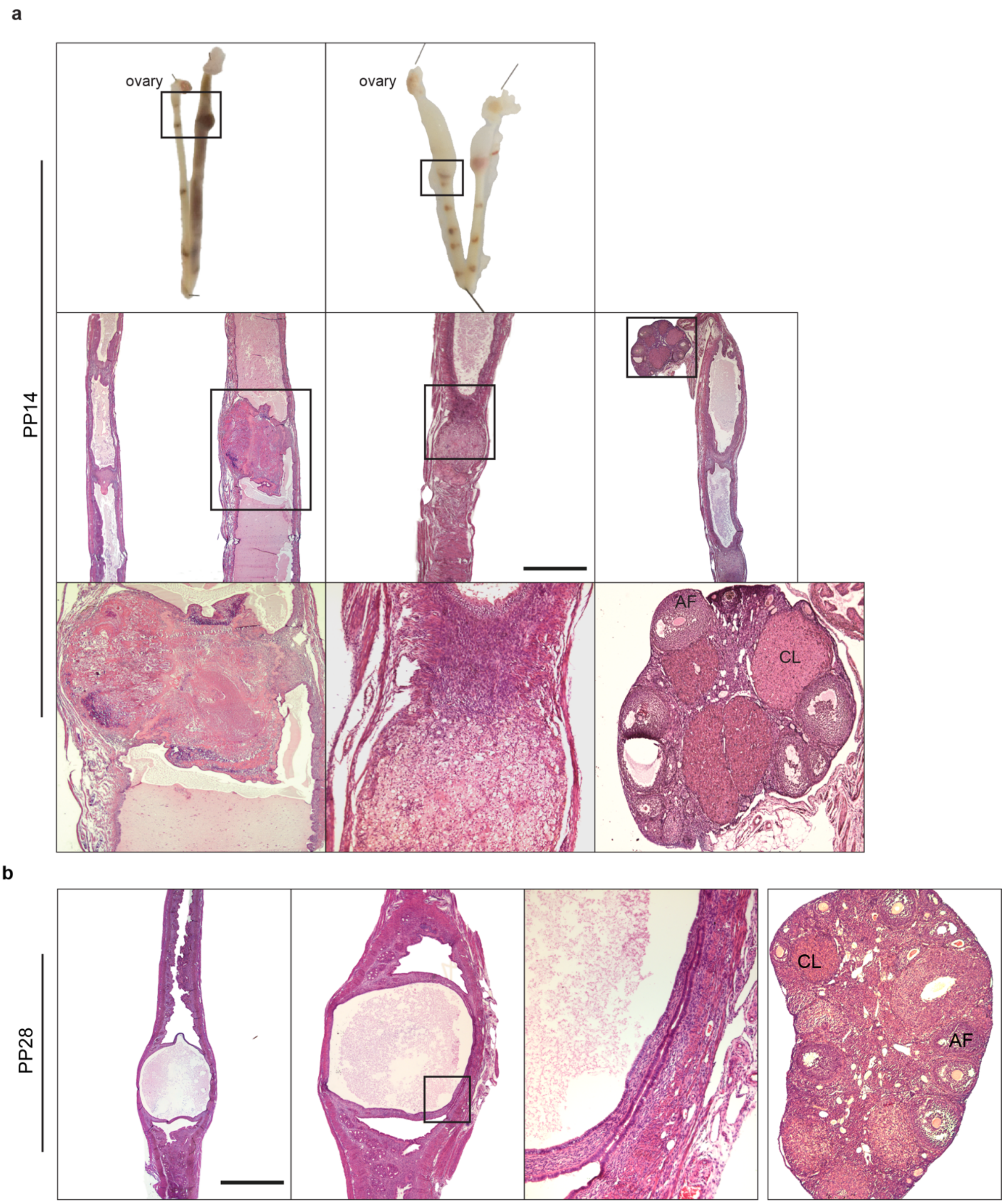

Supplementary Fig. 2 Uterine occlusions persist within Blimp1 mutant uteri at the equivalent 14 and 28 days post-partum. a H&E staining of uteri and ovary at PP14 (n=4). **b** H&E staining of mutant uterus and ovary at PP28 (n=2)**.** Both ovaries displaying follicles at different stages of maturation. AF= antral follicle, CL = corpus luteum. Scale bar = 1mm.

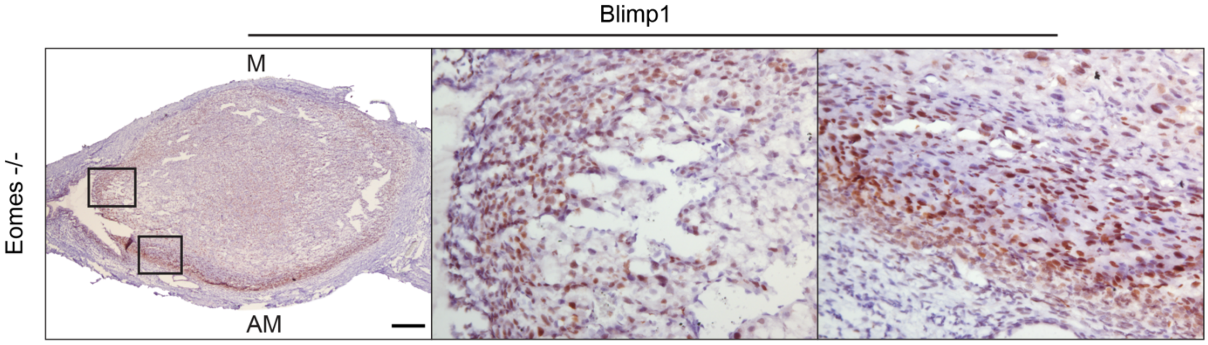

Supplementary Fig. 3 Blimp1 expression at E9.5 in decidua associated with Eomes null embryo. Following embryo death, the decidua fails to expand and Blimp1 is detected at the periphery of the decidual masses undergoing shedding and resorption (n=3 deciduae). M = mesometrial, AM = antimesometrial. Scale bars = 1 mm.

a

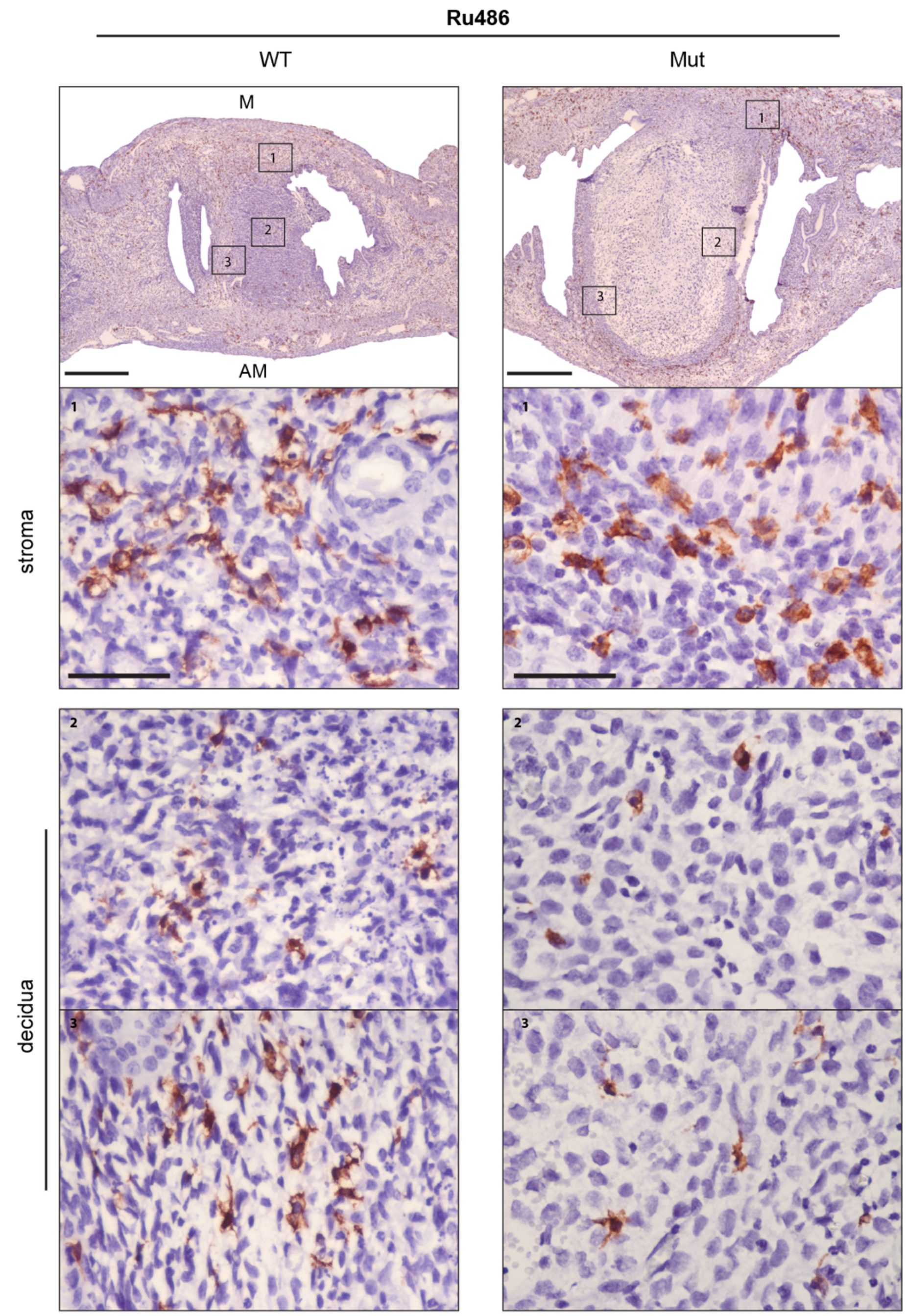
Ru486
WT
Mut
M
AM
1
2
3
stroma
decidua

b

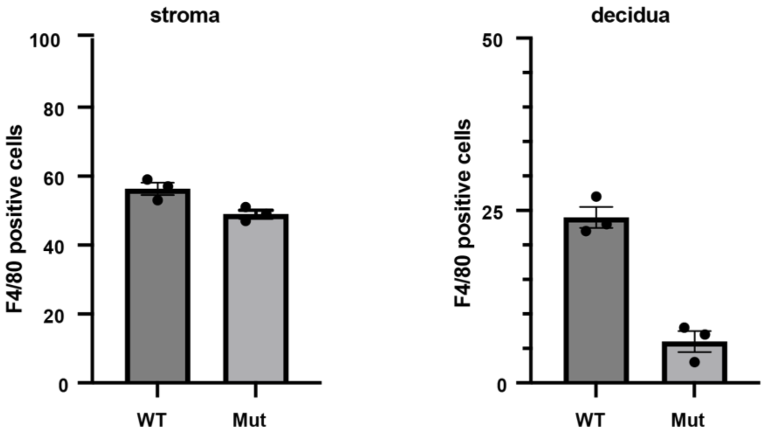
stroma
decidua
F4/80 positive cells
100
80
60
40
20
0
50
25
0
WT
Mut
WT
Mut

Supplementary Fig. 4 F4/80 expression in wild type and mutant uteri 24 hours after RU486 administration at 5.5 dpc. a F4/80 positive macrophages are present in both genotypes (n= 3 deciduae/genotype). Similar numbers of macrophages are present in the uterine stromal cells (box 1). By contrast significantly higher numbers of infiltrating macrophages are present in the wild type deciduae compared to the mutant (box 2 & 3). **b** Quantification of the numbers of F4/80+ cells in the stroma and deciduae of wild type (WT) and mutant (Mut) uteri. Data plotted as the mean of 3 samples per genotype. Error bars represent SEM. Source data are provided as a Source Data file. M = mesometrial, AM = antimesometrial. Scale bars = 500 μm and 50 μm.

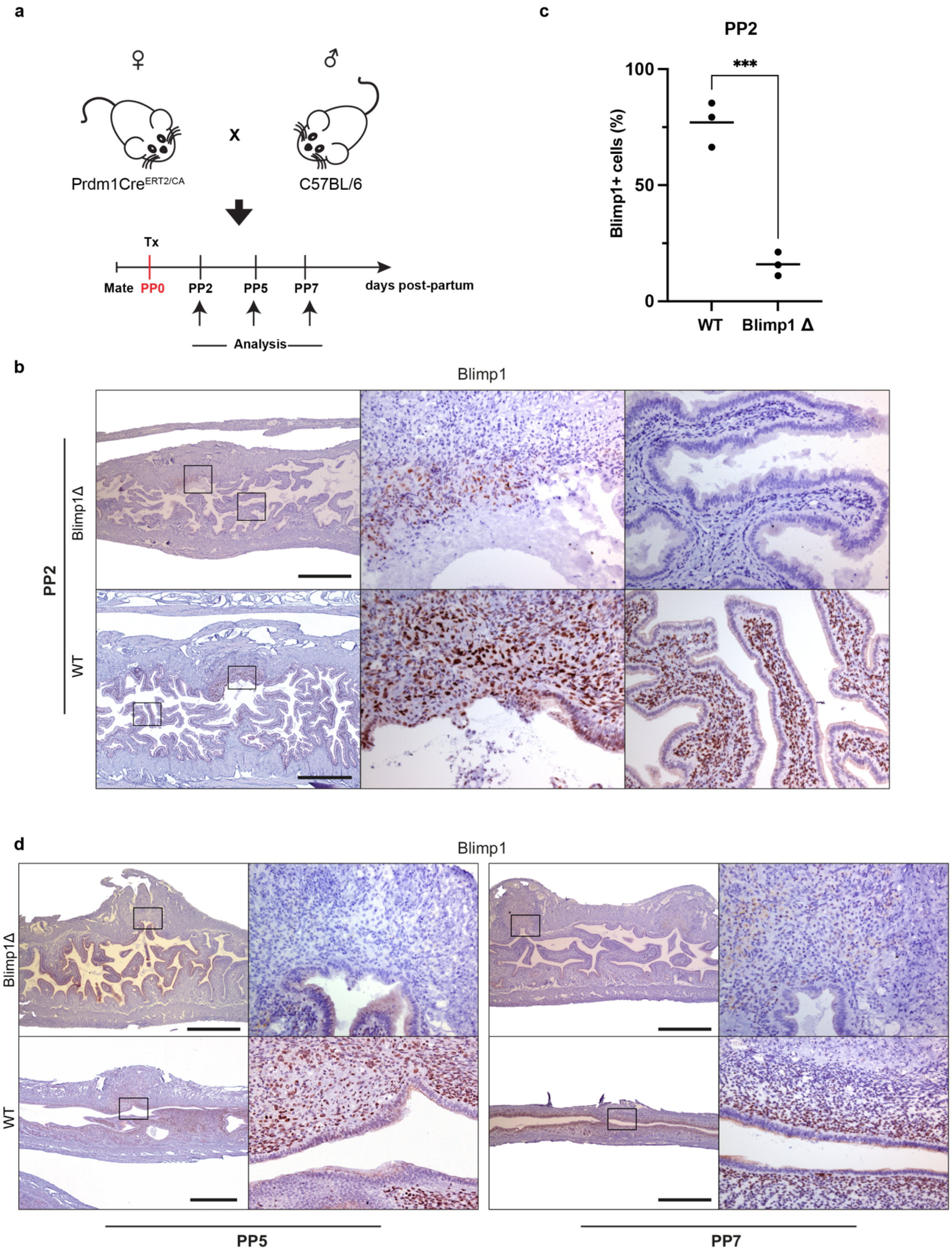

Supplementary Fig. 5 Efficient Blimp1 deletion throughout the uterine stroma of post-partum Blimp1Δ uteri. a *Prdm1Cre $^{ERT2/CA}$* (Blimp1Δ) females were injected with Tx at PP day 0 to inactivate Blimp1 expression. Samples were harvested at PP day 2 (n=5), day 5 (n=3) and day 7 (n=3). **b** Reduced number of Blimp1+ cells in the detachment sites and throughout the endometrium. **c** Quantification of Blimp1+ cells

showing the percentage of reduction of Blimp1 expression in Blimp1Δ samples. Two-tailed unpaired Student's t-test used for the analysis ***$p < 0.001$, $p = 0.0006$. Source data are provided as a Source Data file**. d** At later stages, Blimp1 immunoreactivity is entirely absent in Blimp1Δ females, but increasing in wild type females. Scale bars = 1mm.

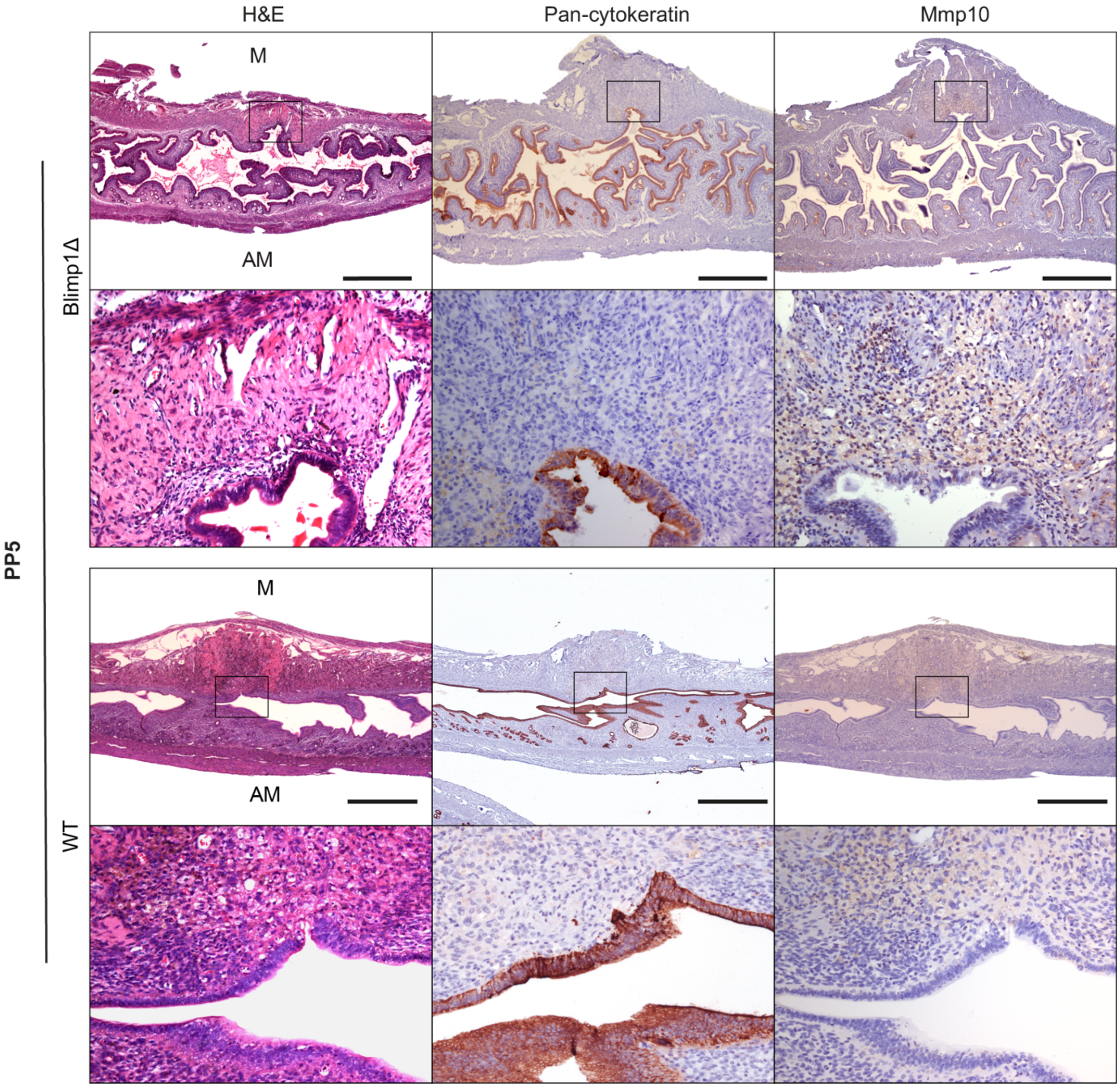

Supplementary Fig. 6 Impaired uterine remodeling at PP day 5 in Blimp1Δ females. H&E staining and expression of p-CK and Mmp10 in mutant (n=3) and wild type (n=3) PP day 5 uteri. M = mesometrial, AM = antimesometrial. Scale bar = 1mm.

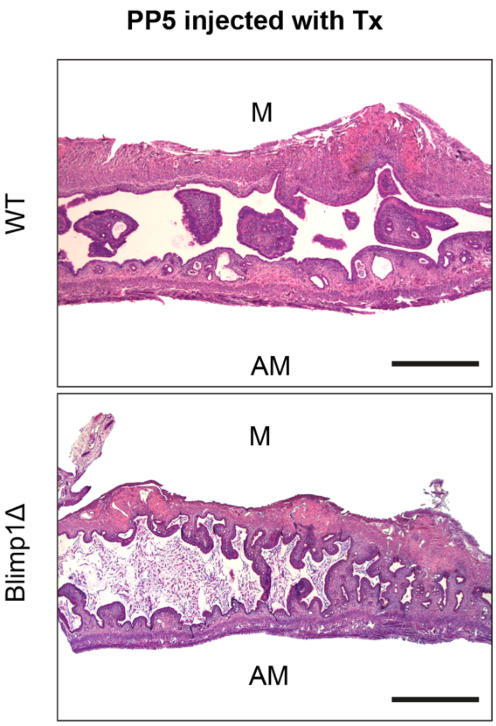

Supplementary Fig. 7 Tamoxifen does not interfere with normal uterus remodeling. A single dose of Tx was injected either to a wild type (n=1) or Blimp1Δ female (n=3) at PP day 5. The wild type uterus undergoes a normal repair process whereas the mutant is disturbed. M = mesometrial, AM = antimesometrial. Scale bar= 1mm.

Antibody	Source	Identifier	Lot	Dilution
Rat monoclonal anti-Blimp1 clone 5E7	Santa Cruz	sc-130917	G9013	1:200
Rabbit polyclonal anti-MMP10	Abcam	ab38930	1003070-1	1:200
Rabbit anti-rat IgG (H+L) - Mouse absorbed	Vector Laboratories	AI-4001	Y0920	1:200
Rabbit anti-Cow cytokeratin	DakoCytomation	Z0622	10122601	1:1000
Rabbit monoclonal anti Phospho-Smad1/5/9 clone D5	Cell Signaling	13820	3	1:500
Chicken polyclonal anti-GFP	Abcam	ab13970	1018753-8	1:1000
Rat monoclonal anti-mouse F4/80	Serotec	MCA497R	1608	1:500
Alexa Fluor 594 donkey anti-rat IgG	Invitrogen	A21209	2041649	1:400
Alexa Fluor 488 Goat anti-chicken IgG	Invitrogen	A11039	1637891	1:400

Supplementary Table 1. Antibodies used for immunohistochemistry and lineage tracing experiments.
