# Supplementary material for: The zinc-finger transcription factor Blimp1/Prdm1 is required for uterine remodelling and repair in the mouse
Source: Nat Commun. 2025 Jan 31;16:1220. doi: 10.1038/s41467-025-56511-8 (PMC11785775; doi:10.1038/s41467-025-56511-8)
Supplement: Supplementary file 2 — Description of Additional Supplementary Files [file 41467_2025_56511_MOESM2_ESM.pdf]

Supplementary Data 1: Related to Figure 5b. Genes differentially expressed in Blimp1 WT decidua (E6.0), 14 hours post RU486 treatment vs Oil controls (DeSeq2 with a FDR cut-off of 0.05, > 2-fold change in expression and FPKM of > 1 in all samples within at least one group). DESeq2 calculates p-values by the Wald test and corrects for multiple testing using the Benjamini and Hochberg (i.e. FDR) by default.

Supplementary Data 2: Related to Figure 5a, 5b. Genes differentially expressed in Blimp1 Mut decidua (E6.0), 14 hours post RU486 treatment vs Oil controls (DeSeq2 with a FDR cut-off of 0.05, > 2-fold change in expression and FPKM of > 1 in all samples within at least one group). DESeq2 calculates p-values by the Wald test and corrects for multiple testing using the Benjamini and Hochberg (i.e. FDR) by default.
